# Optimisation of Sequential Microwave-Assisted Extraction of Essential Oil and Pigment from Lemon Peels Waste

**DOI:** 10.3390/foods9101493

**Published:** 2020-10-19

**Authors:** Antonio Martínez-Abad, Marina Ramos, Mahmoud Hamzaoui, Stephane Kohnen, Alfonso Jiménez, María Carmen Garrigós

**Affiliations:** 1 Department of Analytical Chemistry, Nutrition & Food Sciences, University of Alicante, ES-03690 San Vicente del Raspeig, Alicante, Spain; antma@ua.es (A.M.-A.); marina.ramos@ua.es (M.R.); alfjimenez@ua.es (A.J.); 2 Biomass Valorisation Platform, Celabor scrl, Avenue du Parc 38, 4650 Herve, Belgium; Mahmoud.Hamzaoui@celabor.be (M.H.); Stephane.Kohnen@celabor.be (S.K.)

**Keywords:** lemon waste, pigments, essential oils, microwave-assisted extraction, antimicrobials

## Abstract

In this work, a cascade approach to obtain different valuable fractions from lemon peels waste was optimised using microwave-assisted processes. Microwave-assisted hydrodistillation (MAHD) with a Clevenger apparatus was firstly used to obtain the lemon essential oil (LEO). The remaining residue was then submitted to microwave-assisted extraction (MAE) to extract the lemon pigment (LP). A Box–Behnken design was used to evaluate the influence of ethanol concentration, temperature and time in LP extraction in terms of extraction yield and colour intensity. Optimal extraction conditions for LP were 80% (*v*/*v*) ethanol, 80 °C and 50 min, with a liquid-to-solid ratio of 1:10. The obtained yields for LEO and LP were around 2 wt.% and 6 wt.%, respectively. The composition of LEO was analysed by gas chromatography with flame ionisation detection (GC-FID), and limonene (65.082 wt.%), β-pinene (14.517 wt.%) and γ-terpinene (9.743 wt.%) were mainly identified. LP was purified by using different Amberlite adsorption resins (XAD4, XAD7HP and XAD16N), showing XAD16N the best adsorption capacity. Enrichment factors of 4.3, 4.5 and 5.0 were found for eriocitrin, diosmin and hesperidin, respectively, which were detected as the main components in LP by ultra-high-performance liquid chromatography–diode array detector–tandem mass spectrometry (UPLC-DAD-MS) analysis, with final concentrations of 4.728 wt.%, 7.368 wt.% and 2.658 wt.%, respectively. Successful antimicrobial capacity against *Escherichia coli* and *Staphylococcus aureus* was obtained for LEO. The results from this work showed the potential of applying a cascading approach based on microwave-assisted processes to valorise lemon wastes, obtaining natural pigments and antimicrobials to be applied in food, cosmetic and polymer industries.

## 1. Introduction

Agricultural and food industries produce a large amount of residues and by-products that should be valorised to accomplish the environmental challenges required by the new concepts of circular economy. In particular, citrus fruits are primarily used in juice production, generating a huge amount of residues (mainly peels and pulp) that are very often discarded or burned. These residues show noticeable amounts of potentially valuable chemicals, such as volatiles, pigments and essential oils (EOs), all of them located on the exocarp and flavedo of citrus peels [1,2]. These parts of lemon fruits contain oil glands with specific terpenoids that confer the characteristic lemon fragrance. It is known that EOs are volatile, soluble in organic solvents but insoluble in water at room temperature [3], and they can be used as flavours and aromatic ingredients in food preservatives and cosmetics or therapeutic agents in biomedical applications [4]. Besides, different terpenoids mainly present in EOs (monoterpenes such as citral, pinene, or terpinene, as well as poly(methoxyflavones) and furanocoumarins), have been used since ancient times to treat a variety of health diseases, such as spasms, fever, respiratory problems, cardiovascular diseases, gastrointestinal problems or anxiety [5]. Several works have demonstrated the antimicrobial performance of EOs against pathogenic bacteria and fungus strains [6,7,8,9].

Citrus peels contain two types of natural pigments with differing polarities, such as lipid-soluble carotenoids and water-soluble yellow pigments, which can be a valuable natural source of colourants to replace synthetic ones, providing additional colouring to food products [10,11]. The lemon flavedo contains carotenoids, either xanthophylls (oxygen-containing carotenoids) or carotenes (oxygen-free carotenoids) [12]. Xanthophylls are those compounds that mainly mark the typical yellow colour in lemon peels [13]. Some authors have studied, simultaneously, the phenolic and pigment composition of lemon peels while reporting their beneficial health effects [14]. Eriocitrin, a flavanone present in the flavedo and well-detected in lemon extracts (1002 ± 65.2 mg/kg dry weight), is the main responsible for the extract colouration, together with some carotenoids [13].

Therefore, peels discarded from lemon juice production can be valorised to extract potentially useful compounds, such as the EO and the pigment, resulting in an economic and environmental approach, considering that up to 50–65% of the whole fruit remains as peel by-product [14]. The isolation of EOs from plants or vegetable residues has been traditionally carried out by hydrodistillation (HD) or cold-press, while the pigments have been often obtained by maceration extraction [15]. Nevertheless, microwave-assisted extraction (MAE) can be used in combination with hydrodistillation (MAHD) to isolate valuable constituents from plants [16]. MAE offers some advantages over conventional extraction methods in terms of higher yield and quality of extracts. In addition, the use of MAE reduces considerably the extraction time and the solvent consumption [17]. MAHD was used to obtain the EO from lemon verbena leaves, improving the selectivity of oxygenated compounds, such as citral isomers, compared to conventional HD. Besides, the antioxidant and antifungal activities against nine species of fungi isolated from contaminated Feta cheese were evaluated, obtaining successful results in terms of growth inhibition [5]. Dao et al. obtained a yield of 0.76 wt.% for the extraction of the EO from lemon leaves using MAHD [3].

To the best of our knowledge, no studies have been reported dealing with the use of a cascade approach for the valorisation of lemon peels waste to extract sequentially different fractions using microwave-assisted methods, such as the EO and the pigment, and further monitoring of their antimicrobial activity. So, this work aims to investigate the extraction of (i) the EO from lemon peels by MAHD, followed by the extraction of the residual sample to obtain (ii) the lemon pigment by MAE. For the lemon pigment, MAE experimental parameters were optimised through response surface methodology (RSM) based on a Box-Behnken design (BBD) in terms of total yield and colour intensity. The obtained pigment was further purified by using adsorption resins. Finally, the main compounds present in lemon EO and pigment were determined by gas chromatography with flame ionisation detection (GC-FID) and ultra-high-performance liquid chromatography–diode array detector–tandem mass spectrometry (UPLC-DAD-MS), respectively. The obtained results of this study could support the comprehensive utilisation of citrus fruits waste to obtain bioactive compounds to be further applied in different industrial sectors, including food products.

## 2. Materials and Methods

### 2.1. Materials and Reagents

Lemon waste was obtained from discarded whole fruits, due to inadequate appearance or calibre, from Federación de Cooperativas Agrarias de Murcia (FECOAM, Murcia, Spain). The flavedo was mechanically peeled and slightly cut into small pieces by using a household blade cutter for 10–20 s at medium speed to reach particles of 1–50 mm^3^. All chemicals used in this work were of analytical grade, and they were purchased from Sigma-Aldrich (Madrid, Spain).

### 2.2. Microwave-Assisted Extraction (MAE)

MAE was performed using a FLEXIWAVE™ microwave oven (Milestone srl, Bergamo, Italy). A sequential extraction process was optimised, consisting of a first extraction of the lemon essential oil (LEO) followed by a subsequent extraction of the lemon yellow pigment (LP).

For LEO, MAHD was used with a Clevenger-type apparatus. Seven hundred and fifty grams of lemon waste were used for extraction at two different water-to-solid ratios (1 and 0.3 mL/g). The MAHD process was divided into a first step with the sample heated near to the boiling temperature and a second step where the oil was distilled. Irradiation power and extraction time conditions were selected according to previous optimisation tests, and they are shown in Table 1. The collected oil phase was centrifuged at 4000 *g* for 10 min to separate water and oil phases, obtaining a transparent LEO extract. Extractions were performed in triplicate.

After LEO extraction, LP was subsequently extracted without any intermediate steps. Six grams of the remaining solid waste obtained after LEO hydrodistillation were introduced into a 250-mL round-bottom flask and mixed with the extraction solvent (depending on the predefined experimental conditions) at a liquid-to-solid ratio of 1:10. Samples were heated in the microwave oven and extracted by controlling time and temperature for each experiment. Microwave power (500 W), stirring rate (400 rpm) and heating rate (20 °C/min) were kept constant during the MAE process. Extracts were then filtered, and polysaccharide compounds were precipitated by adding 96% (*v*/*v*) ethanol. Samples were kept overnight in a freezer at −20 °C, and they were vacuum-filtered. The ethanol present in the samples was then removed in a rotary evaporator (R-300, Büchi Labortechnik AG, Flawil, Switzerland), and the aqueous solution was freeze-dried (LyoQuest Plus, Telstar, Terrassa, Spain). The obtained extract was stored in the darkness at room temperature until further analysis.

Response surface methodology (RSM) was used to determine the optimal extraction conditions of LP. The effects of three extraction variables (ethanol concentration, temperature and extraction time) were investigated using a Box-Behnken experimental design (BBD). Table 2 shows the selected extraction variables and their levels, which were set according to the related bibliography, experimental limitations and previous tests. This design consisted of 15 experiments that were performed randomly and are listed in Table S1 (Supplementary Materials). Three central points were added to estimate the model’s pure error. The responses obtained from the experimental design were evaluated in terms of overall extraction yield and colour intensity.

A multiple linear regression analysis was performed to obtain the regression coefficients following a second-order polynomial model:

(1) $$Y=\beta_{o}+\sum \beta_{i}X_{i}+\sum \beta_{ii}X_{i}^{2}+\sum \beta_{ii}X_{i}X_{i}$$

where *Y* is the predicted response; *X_i_* and *X_j_* are the actual values of the independent variables; *β_o_* is a constant and *β_i_*, *β_ii_* and *β_ij_* are the regression coefficients for the linear, quadratic and interactive effects of variables *i* and *j*, respectively.

### 2.3. Characterisation of MAE Extracts

#### 2.3.1. Extraction Yield

The overall extraction yield (wt.%) was calculated and used as the response in the BBD using the following equation:

(2) $$\mathrm{Extraction}\;\mathrm{yield}\;\left(\mathrm{wt}.\%\right)=\frac{W_{\mathrm{ext}}}{W_{0}}\cdot 100$$

where *W*_ext_ is the weight of the extracted material (g), and *W*_0_ is the weight of raw material used before extraction (g).

#### 2.3.2. Colour Intensity

The colour intensity was measured by dissolving the freeze-dried LP extract in 5 mL of 70% (*v*/*v*) ethanol, and an aliquot was further diluted until absorbance values ranging between 0.2–0.8 could be taken at 440 nm by using a Biomate-3 UV-VIS spectrophotometer (Thermospectronic, Mobile, AL, USA).

### 2.4. Purification of LP

LP extracts obtained by MAE were further purified by using three different adsorption resins with different polarity: Amberlite XAD4, Amberlite XAD7HP and Amberlite XAD16N. The physical properties of these resins are summarised in Table 3. The screening and selection of the best resin was evaluated based on its adsorption (retention) power of the coloured metabolites. The quantification of the adsorption strength was carried out by measuring the absorbance of the extract before and after the treatment with the resins using a UV-Vis spectrophotometer at 440 nm.

Two grams of the LP extract were firstly dissolved in 1 L of demineralised water and loaded slowly through a glass column containing 20 g of the adsorption resin previously conditioned with water and ethanol, as recommended by the producer. After the loading step (around 30 min), 1 L of water was passed through the column to ensure the complete elution of the undesired compounds (sugars, pectin, amino acids, polysaccharides, oligosaccharides, etc.). Then, 250 mL of absolute ethanol (99%, *v*/*v*) were used for the desorption step, which consisted in the recovery of the retained metabolites (polyphenols, flavonoids, phenolic acids, etc.) from the macroporous resin thanks to the solubilisation in an organic solvent, ethanol. After that, the resin was washed with water and stored in 50% (*v*/*v*) ethanol for further use. The recovered ethanol fraction was then evaporated, and the aqueous residual fraction was freeze-dried. The LP-rich fraction was stored in the darkness at room temperature until further analysis.

### 2.5. Characterisation of LEO and LP

#### 2.5.1. GC-FID Analysis of LEO

The analysis of the main volatile constituents present in LEO was performed, in triplicate, using a Shimadzu GC 2014 system (Shimadzu, Kyoto, Japan) coupled to a FID detector and equipped with a RTXi-5ms column (30 m × 0.25 mm × 0.25 μm). The carrier gas was helium, with a flow rate of 1.25 mL/min. The oven temperature program used was: 50 °C (0.5 min) to 120 °C at 20 °C/min and then raised to 240 °C at 5 °C/min (5 min). The injector and detector temperatures were set at 285 °C and 300 °C, respectively. One microliter of LEO diluted 100× (*v*/*v*) in hexane was injected with a split ratio of 1:20. The main LEO components were identified by their Kovats retention index, and their relative concentrations were obtained by peak area normalisation.

#### 2.5.2. UPLC-DAD-MS Analysis of LP

UPLC analysis of LP before and after purification was performed, in triplicate, using a Waters UPLC system coupled with a Xevo TQ-S detector (Waters Corporation, Milford, MA, USA). Twenty milligrams of LP-rich fraction were dissolved in 10 mL of MeOH:DMSO 9:1 (*v*/*v*) and passed through a 0.22-µm poly(tetrafluoroethylene) (PTFE) filter. Quantification of the major polyphenolic compounds was performed based on integrated peak areas of samples and standards diluted in MeOH:DMSO 9:1 (*v*/*v*) using external calibration (1–10 mg/kg). Chromatographic separation was performed with an ACQUITY UPLC Shield RP18 (100 mm × 2.1 mm × 1.7 µm) (Waters Corporation, Milford, MA, USA) at 40 °C. The mobile phase was composed of two solvents: water containing 0.6 wt.% formic acid and 126-mg ammonium formate (A) and acetonitrile (B). The linear gradient used was: 5% B (7.2 min) to 20% B (10 min) to 50% B (12 min) to 100% B. The flow rate was set to 0.5 mL/min, and the injection volume was 3.5 μL. Electrospray positive or negative ionisation mode was used depending on the targeted molecule. Desolvation was carried out using a nitrogen gas flow (800 L/h) and high temperature (550 °C). Cone voltage was set at 3 kV. Specific multiple reaction monitoring (MRMs), cone and collision voltage were used for each compound.

#### 2.5.3. Antimicrobial Performance of LEO and LP

Antimicrobial tests for LEO and LP-rich fractions were performed against *Staphylococcus aureus* (*S. aureus*) and *Escherichia coli (E. coli)* as model Gram-positive and Gram-negative bacteria, respectively. A traditional broth macrodilution method was performed, adding the LEO and LP-rich fraction at increasing concentrations to test tubes containing phosphate-buffered saline enriched with tryptic soy broth (TSB, 1 wt.%). This rich medium allowed the proliferation of the tested bacteria around two orders of magnitude in control samples. The tubes were incubated at 35 °C for 24 h, and then, bacteria were enumerated by plate count on tryptic soy agar (TSA). A 10% (*v*/*v*) DMSO solution was used to improve the solubility of the studied extracts (LEO and LP-rich fraction).

### 2.6. Statistical Analysis

Statgraphics Centurion XVI (Statistical Graphics, Rockville, MD, USA) was used to generate and analyse the BBD results. The graphic analysis of the main effects and interactions between variables was used, and the analysis of variance (ANOVA) was carried out. SPSS 15.0 (Chicago, IL, USA) was used to perform the statistical analysis of experimental data by one-way analysis of variance (ANOVA). Differences between average values were assessed based on the Tukey test at a confidence level of 95% (*p* < 0.05).

## 3. Results

### 3.1. MAE Optimisation

A cascade extraction process from lemon waste was developed and optimised, consisting of a first extraction of the essential oil, followed by the extraction of the yellow pigment separately from the same batch in a subsequent process while achieving desirable selectivity levels and yield in both extracts. The MAE process design consisted of adding the suitable solvent for pigment extraction to the extraction vessel directly after obtaining the essential oil using MAHD, without any intermediate steps.

Table 1 shows the extraction yield obtained for LEO according to the two conditions used. A higher energy was needed during the heating step when using a water-to-waste ratio of 1 mL/g due to the higher amount of water present in the extraction vessel. No significant differences were obtained in both methods, showing yield values around 2 wt.%. So, it was decided to use a water-to-waste ratio of 0.3 mL/g in terms of energy saving. Similar yield results were found by other authors but reported significantly longer extraction times. Ben Hsouna et al. reported a 3 wt.% yield for the EO extracted from flesh flowers of *Citrus limon* by conventional HD during three h using a Clevenger-type apparatus [7]. Yield (2.47 wt.%) was obtained by Hien Tran et al. when using MAHD at 2.80-mL/g water-to-material ratio for 63.29 min [18]. Moreover, although an extraction time of 10 min was set in this work to ensure most of the essential oil was extracted, it was observed that higher than 90% of the obtained yield was extracted after four min of hydrodistillation. A similar behaviour was reported by Golmakani et al. [19], who observed that increasing extraction times by MAHD did not lead to significant increases in essential oil yields from lemon peels, which were, in all cases, lower than 1.4 wt.% on a dry basis. So, the MAHD method proposed in this work offers important advantages over previous reported studies in terms of lower extraction time, being a green and rapid method to efficiently and cost-effectively extract LEO from lemon wastes.

The use of MAHD compared to HD for extracting essential oils from other natural sources such as orange peels (*Citrus auranticum* L.) and mango (*Mangifera indica* L.) flowers has been also reported [20,21]. Authors suggested that the use of microwaves results in a more efficient heating up of the inner part of the tissue, causing a quick rupture of the glandular walls, improving the release efficiency of the essential oil from the food matrix at shorter times.

After LEO extraction, a BBD was used to optimise the MAE of LP from the remaining solid waste consisting of 15 experiments, including three central points. The design matrix and results obtained for all experiments are given in Table S1. The influence of ethanol concentration, temperature and extraction time was evaluated on two response variables (extraction yield and colour intensity). These responses were selected in order to determine the final amount and colour properties of the extracted LP. Yields ranging from 3.2–6.2 wt.% were obtained, showing experiments 2 and 4 as the lowest and highest yields, respectively. Regarding colour intensity, the lowest and highest values were observed for experiments 2 and 13, respectively.

The obtained experimental responses were fitted using a second-order polynomial model giving Equations (3) and (4), which predicted the optimal conditions for maximising the two studied responses, independently.

Yield = 10.3825 − 0.030463 A − 0.189678 B − 0.0429339 C − 0.000037037 AA + 0.000708333 AB + 0.000333333 AC + 0.00135417 BB − 0.000272727 BC + 0.000914601 CC(3)

Colour intensity = 1.11233 – 0.0045197 A − 0.0230761 B – 0.000839394 C + 0.00000791667 AA + 0.00012125 AB − 0.0000293939 AC + 0.000136562 BB + 0.0000677273 BC − 0.0000236364 CC(4)

where A, B and C correspond to the ethanol concentration, temperature and extraction time, respectively.

Analysis of variance (ANOVA) was performed to evaluate the effects of the extraction variables in the studied responses, as well as the reliability of the fitted models (Table S2 in the Supplementary Materials). The R^2^ values obtained for the studied models (0.8675 and 0.8820 for yield and colour intensity, respectively) indicated a good degree of correlation between the experimental and predicted values. Moreover, the nonsignificance (*p* > 0.05) of the lack-of-fit tests (with *p*-values of 0.1022 and 0.1530 for yield and colour intensity, respectively) verified the good fitness of the proposed models.

**Figure 1:**
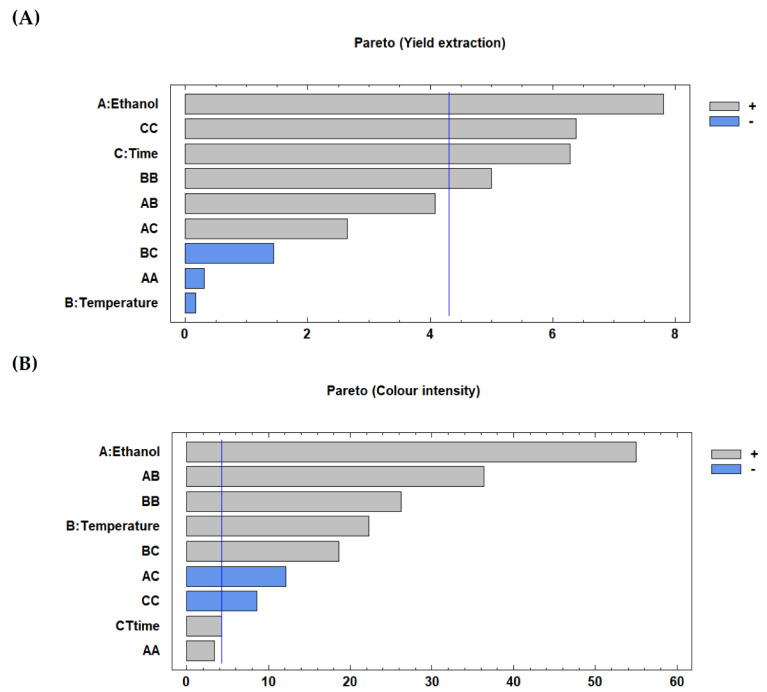
Pareto charts obtained for the extraction yield and colour intensity of lemon pigment (LP). Extraction yields (**A**); colour intensity (**B**).

Figure 1 shows Pareto charts and significant effects at 95% confidence obtained for the studied responses. The extraction yield (Figure 1A) was significantly affected by the ethanol concentration, time and temperature (quadratic interaction), with a positive effect. Regarding colour intensity (Figure 1B), it was positively influenced by the ethanol concentration followed by extraction temperature. Significant positive interactions between ethanol concentration-temperature and temperature-time also affected the colour intensity, increasing this response with higher values of the mentioned variables. In contrast, a significant negative interaction between ethanol concentration-time was observed, together with a negative quadratic interaction for time.

Both responses (yield and colour intensity) were positively affected by the ethanol concentration, as it is well-known that the solvent concentration is one of the most relevant factors to be considered when designing a MAE method [22]. A good selection of the solvent allows a good solubility of the target compounds, as well as solvent penetration, enhancing the extraction of the compounds from the matrix. In addition, the solvent selection will influence the dielectric constant and the mass transfer kinetics of the MAE process [23]. Csiktusnádi Kiss et al. optimised the MAE conditions for pigments extraction from paprika (*Capsicum annuum* L.) powders, demonstrating that the calculated dielectric constant of the solvent exerted a significant influence both on the strength and selectivity of the extraction [24]. Besides, Kaderides et al. suggested that the extraction yield should increase with an increase in the solvent polarity [25]. Other authors have reported that high percentages of ethanol can break the hydrogen and hydrophobic bonds existing between phenolics–proteins and phenolics–cellulose in ethanol/water solutions, improving the extraction process [26]. However, some authors have demonstrated that the presence of small amounts of water in the solvent during MAE could favour a possible diffusion of water into the matrix cells, achieving a better heating and, thus, helping the transfer of compounds into the solvent at higher mass transfer rates [27].

Regarding extraction temperature, higher values positively influenced both responses, in particular, the colour intensity. The use of temperature values close to the boiling point of the solvent could speed up intermolecular interactions and facilitate molecular movement, increasing the solubility of the solutes into the solution [28]. However, it should be also considered that high temperatures may cause a decrease in LP content, probably due to a rearrangement of the molecules or its own thermooxidation [24].

**Figure 2:**
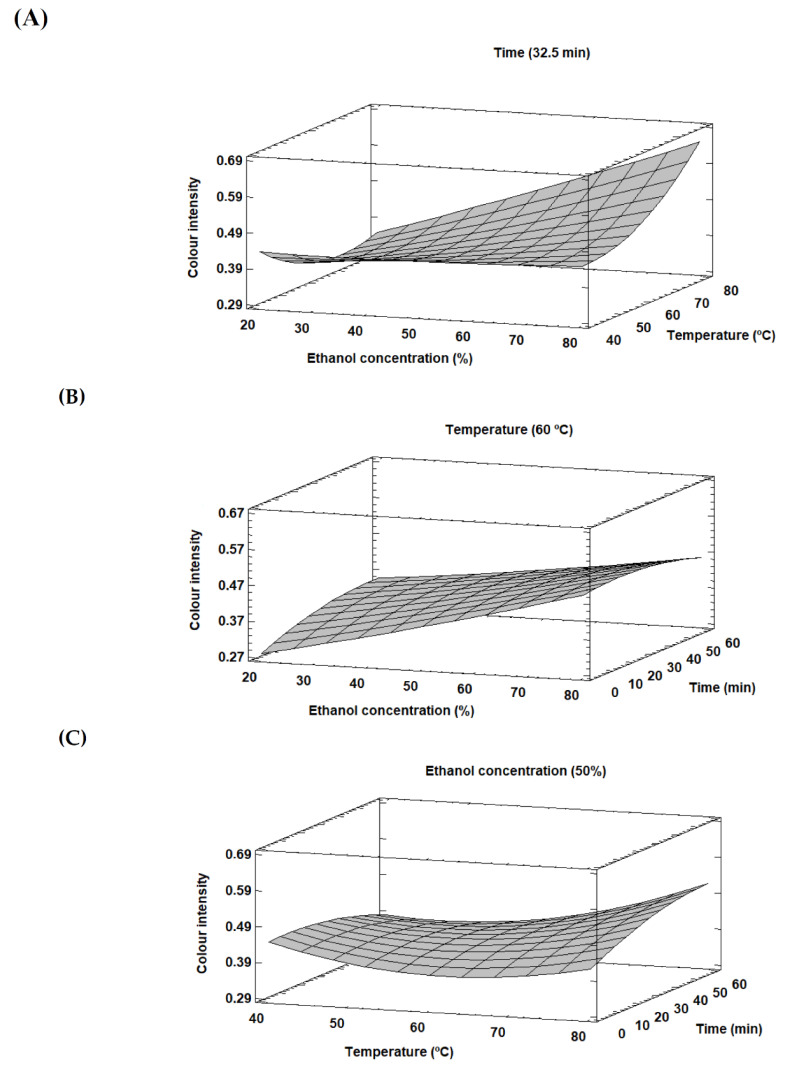
Response surface plots showing the effect of the studied variables on the colour intensity for LP. Interactions between ethanol concentration-temperature (**A**), ethanol concentration-time (**B**) and temperature-time (**C**).

A graphical analysis in terms of response surface plots was carried out for colour intensity (Figure 2) to better evaluate the interactions present between the studied extraction variables on this response, giving valuable information for the analysis and optimisation of the extraction conditions [29]. Interactions between ethanol concentration-temperature, ethanol concentration-time and temperature-time are presented in Figure 2A–C, respectively. As it can be seen in Figure 2B,C, long extraction times are needed to obtain high response values. Regarding temperature, this variable clearly affects the response by increasing the solubility and diffusion coefficients of the target compounds and decreasing the solvent viscosity [25]. In this sense, temperatures up to 60 °C should be applied to produce a positive effect in the response. At fixed temperature values, the time and ethanol concentration must be increased to increment the response. Moreover, a high ethanol concentration is needed to maximise the response, since a minimum amount of ethanol fraction is necessary to contribute to the destruction of cell walls, movement of the extraction solvent and diffusion inside the cells [29]. This behaviour could be related to the change in solvent polarity with the ethanol addition and the high solubility of polyphenols in organic solvents [30].

The optimal MAE conditions for LP were obtained by simultaneously maximising the extraction yield and colour intensity by using a desirability function to search a combination of the different variable levels that satisfy simultaneously all the requirements for each response [31]. A desirability value of 0.990 was obtained with optimal extraction conditions of 80% (*v*/*v*) ethanol, 80 °C and 50 min. Validation tests performed under these optimal conditions, in triplicate, resulted in experimental values of 5.9 ± 0.3 wt.% for the extraction yield and 0.669 ± 0.004 for the colour intensity. These values did not differ significantly with the obtained predicted values by the models of 6.2 wt.% and 0.657 for extraction yield and colour intensity, respectively. Therefore, it was concluded that the developed quadratic models (Equations (3) and (4)) were reliable to optimise the MAE of LP extracted from lemon peels within the range of the independent variables studied.

MAE has been proposed as a very promising method for natural colourants extraction in different food products, being characterised by a typical two-step diffusion process: an initial extraction from the exterior of the cells, followed by the diffusion of the solute across the membranes [32]. MAE has been applied for the recovery of different natural pigments, such as anthocyanins, curcumin, carotenoids, safflower yellow, flavonoids, safflomin A and lycopene, among others [33]. Regarding lemon pigment, Chen et al. obtained a highly stable and water-soluble food dye called yellow #15 from the ethanol extract of lemon peels by sonication [14]. However, no study has been found reporting MAE for the extraction of yellow pigments from lemon peels.

### 3.2. Identification of Major Phenolic Compounds Present in LEO

The composition of LEO was investigated by GC-FID. A total of twenty-one chemical compounds were identified (Table 4) from the EO fractions. Six major compounds were found in LEO: α-pinene (1.992 wt.%), sabinene (2.395 wt.%), β-pinene (14.517 wt.%), myrcene (1.427 wt.%), limonene (65.082 wt.%) and γ-terpinene (9.743 wt.%). Other minor components (<1 wt.%) were also identified, as shown in Table 4. These compounds can be quickly evaporated at room temperature due to their volatility, and they are responsible for the characteristic aroma present in LEO. The results obtained in this work are in agreement with previous chemical compositions identified by other authors [7,18], who reported the presence of alkaloids, mono and sesquiterpenoids, acyclic and cyclic hydrocarbons, phenolic and polyphenolic derivatives and their oxygenated derivatives [6,34,35,36]. EOs composition and concentration of ingredients depend on different factors, such as geographical distribution; citrus variety and environmental conditions such as temperature, precipitations, hours of sunshine and altitude [34].

Limonene has been reported to be the major chemical constituent present in lemon EOs. Shakir et al. reported [37] concentrations of limonene of 65.2867 wt.% and 59.156 wt.% in the EO obtained from lemon *(Citrus limon)* peels by HD and MAHD, respectively, in accordance with our results (65.082 wt.%) but using longer extraction times. Yazgan et al. [38] found a similar limonene concentration of 52.85 wt.% in lemon (*Citrus limonum*) plant EOs by HD using an industrial type of Clavenger device for four h. Dao et al. [36] reported α-citral as the main component found in EO from lemon (*Citrus aurantifolia* L.) leaves by MAHD, reaching a content of 27.982 wt.%, followed by β-citronellol and D-limonene at 20.06 wt.% and 15.732 wt.%, respectively.

Some major ingredients present in LEO, such as α-pinene, β- pinene, β-myrcene and limonene, have been reported to be used in the medical industry as anaesthetics and antiseptics and for the preparation of perfumes and flavouring agents. Therefore, the obtained LEO could be a potential additive to be applied in cosmetic, food or medical sectors [9,39].

### 3.3. Purification and Characterisation of LP

#### 3.3.1. LP Purification with Amberlite Adsorption Resins

**Figure 3:**
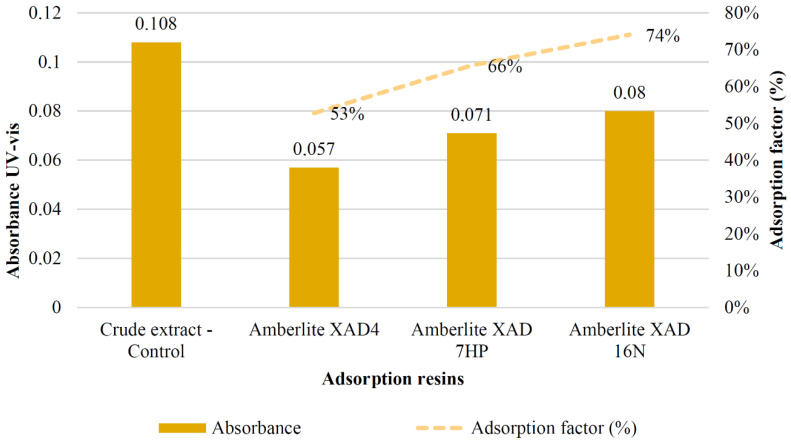
Adsorption profile in absorbance by ultraviolet-visible spectroscopy (UV-Vis) of LP extract by using macroporous resins with different polarity.

The enrichment of the LP colouring metabolites extracted by MAE was studied by using adsorption macroporous resins. These resins present some advantages compared to other separation and enrichment methods, such as their relative low cost, ease of use and the possibility to be regenerated. Besides, these resins have high efficiency, and they are suitable for industrial process scale-up [40]. The affinity of the targeted molecules for the macroporous resin depends on its physicochemical properties (hydrophobicity, polarity, pore diameter, surface area, particle size, etc.). The results obtained for the adsorption profile of the targeted molecules in LP by using different adsorption resins are given in Figure 3. As it can be seen, the minimum adsorption power/strength was found when using Amberlite XAD4 resin with an absorbance value of 0.057, corresponding to an adsorption factor of 53%, compared to the crude LP extract used as the control (absorbance of 0.108). The use of Amberlite XAD7HP resin (absorbance of 0.071) achieved an adsorption factor of 66%. Finally, the best result was found when using Amberlite XAD16N with an absorbance value of 0.08, corresponding to an adsorption factor of 74%. Based on these results, Amberlite XAD16N was selected for the fractionation experiments.

The use of Amberlite resins has been previously reported as an efficient adsorption-desorption purification method. Ferreira-Dias et al. studied the removal of different compounds (pigments, free fatty acids and oxidation products) from olive residue oil miscella by using the same three resins applied in this work (Amberlite XAD4, XAD7HP and XAD16N), being the adsorption efficiency of every group of compounds dependent on the adsorbent and the adsorbent/oil ratio used [41]. Coutinho et al. [42] used adsorption-desorption processes on Amberlite XAD7HP and Sephadex LH20 resins to partially purify anthocyanins present in the juice of red cabbage, showing the Amberlite resin had a higher adsorption capacity compared to Sephadex LH20.

The obtained results are in good agreement with those obtained by Sandhu and Gu, who studied the adsorption/desorption properties of anthocyanins from muscadine (*Vitis rotundifolia*) juice pomace water extracts on five different Amberlite resins [43]. These authors found that the adsorption capacity was highest on nonpolar resins (FPX-66 and XAD16N), showing XAD-7HP (polar) had the lowest adsorption capacity and ratio. This effect was attributed to strong interactions between polar hydroxy groups of anthocyanins in the solute with the adsorbent material. In addition, XAD16N has a higher surface area than XAD7HP, with values of 800 and 500 m^2^/g, respectively (Table 3). The pore size of the adsorbents and the size of adsorbate molecules also play an important role in the adsorption process, showing too-small pore diameters (such as XAD4), a restriction in the diffusion of adsorbate molecules, whereas too-large pore diameters could favour the desorption of the adsorbed molecules [43]. These differences in polarity, surface area and pore size could favour the adsorption of the phenolic compounds present in LP on the adsorbent phase, which contain polar hydroxyl groups and nonpolar groups, explaining the differences found in the adsorption behaviour shown in Figure 3. However, the adsorption of a solute on an adsorbent is a complex process that requires the interaction between three components: the adsorbent, the adsorbate or solute and the solvent, involving a physical action through hydrogen bonding or van der Waals forces [43]. As a result, the XAD16N nonpolar resin with a large surface area and ideal pore diameter (Table 3) showed the higher adsorption capacity for LP.

The use of food-grade adsorbents for the purification and fractionation of phenolic compounds from natural extracts could be a valuable technique to be studied at the pilot scale, having the obtained purified fractions’ potential functional applications, such as food ingredients or dietary supplements [40,44].

#### 3.3.2. Characterisation of Purified LP Fraction

**Figure 4:**
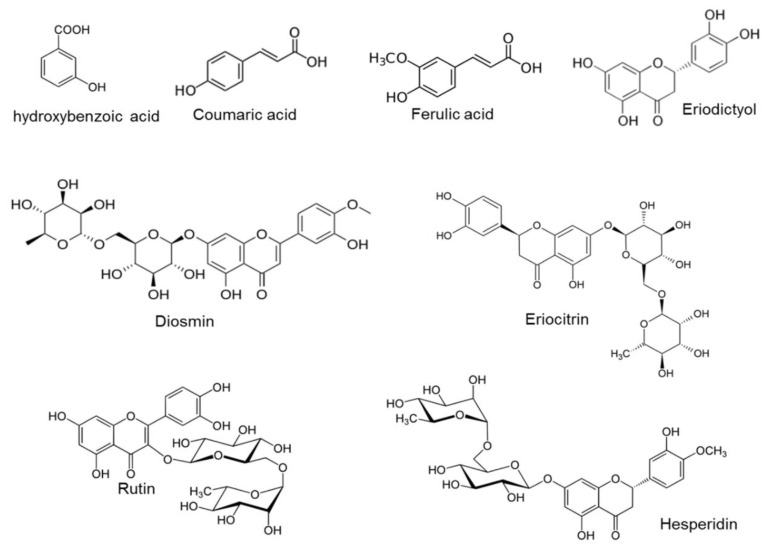
Major phenolic acids and flavonoids found in the crude LP extract.

The obtained crude LP extract by the MAE and LP-rich fraction after purification using Amberlite XAD16N resin were characterised by UPLC-DAD-MS, and major polyphenolic constituents were identified and quantified. Three main compounds were identified, including hesperidin, eriocitrin and diosmin (Figure 4). Other minor components such as ferulic acid, rutin, hydroxybenzoic acid, eriodyctiol and coumaric acid were also found.

**Figure 5:**
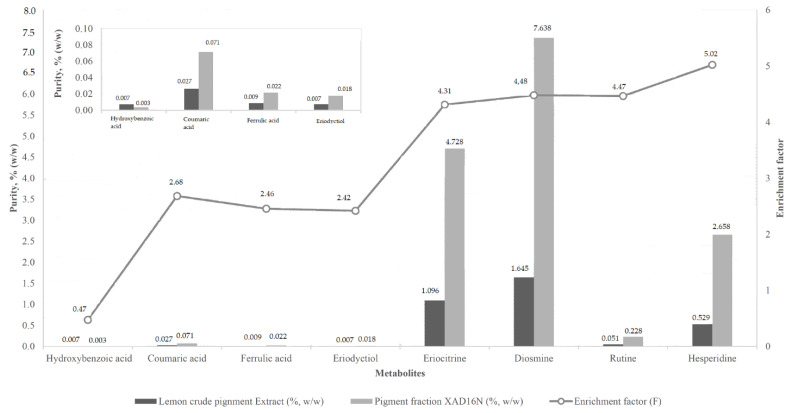
Comparison of LP extract purity before and after fractionation with Amberlite XAD16N resin. The enrichment factor (F) is given by the secondary axis.

The results obtained for the quantification of the major phenolic acids and flavonoids present in LP are summarised in Figure 5. As it can be seen, the major compounds found in the crude LP extract were significantly concentrated, and their purity was increased by several folds. Indeed, eriocitrin, diosmin and hesperidin present in the initial extract in a concentration of 1.096 wt.%, 1.645 wt.% and 0.529 wt.%, respectively, were enriched in the final LP-rich fraction after treatment with XAD16N resin, resulting in concentrations of 4.728 wt.%, 7.368 wt.% and 2.658 wt.%, respectively. The obtained enrichment factors for these compounds were 4.3, 4.5 and 5.0, respectively. Based on these three major components, the total LP content in the final purified fraction was around 14.7 wt.%.

Ledesa-Escobar et al. also identified some remarkable flavonoids present in lemon waste extracts obtained by MAE, such as flavanones (hesperidin, eriocitrin or naringin); flavones (diosmin) and flavanols (rutin) [45]. In addition, Di Donato et al. also detected hydroxycinnamic acids such as p-coumaric, ferulic and caffeic acids together with flavonoid compounds [46].

### 3.4. Antimicrobial Performance of Lemon Extracts

The antimicrobial performance of the LEO and LP-rich fractions against two food-borne bacteria was assessed, and the obtained results are presented in Table 5. Regarding LP, no antimicrobial effect against the tested bacteria and LP concentrations was observed, even if the polyphenol compounds that could contribute to provide some antibacterial properties were already identified. These results suggested that LP might not be used as an antimicrobial additive, even at high LP concentrations. However, this extract coming from lemon wastes could be considered a natural yellow pigment to be used as a food colourant [14] or incorporated into laminar nanoclays to be applied in nanocomposites as colouring additives [47] to replace synthetic yellow dyes with naturally derived alternatives.

Concerning LEO, the results of the antimicrobial properties at concentrations ranging from 15–5000 ppm against Gram-positive and Gram-negative strains are also shown in Table 5. Control samples from both bacteria were able to proliferate around two log units after 24 h of incubation, being the initial inoculum sizes of 5.72 ± 0.26 and 5.80 ± 0.01 log colony-forming units (CFU)/mL for *S. aureus* and *E. coli,* respectively. LEO extracts showed some growth inhibition at a concentration of 50 ppm for *S. aureus*, while a bacteriostatic effect was reached by using 150 ppm and 500 ppm for *S. aureus* and *E. coli,* respectively. The death of 99.9% of the inoculated bacteria was achieved at concentrations higher than 1500 ppm in both cultures, resulting in values lower than 1 for *S. aureus* and approximately 1.45 for *E. coli*. The detected antimicrobial activity confirms the efficacy of LEO as a potential natural antimicrobial source against food-borne pathogens to be applied in food systems and the pharmaceutical industry [7,18], in addition to its characteristic fragrance properties.

The antibacterial properties of lemon EOs have been studied by other authors, although few studies were published compared to other natural extracts [48,49,50]. The obtained results were in accordance with previous reported data for lemon essential oil [7,38], showing an inhibitory activity against different target strains by negatively affecting the lag time, growth rate and final growth level. Caputo et al. [51] studied the antimicrobial activity of citrus water extracts obtained by conventional extraction methods and MAE against ten different sanitary relevant bacteria. They found that MAE extracts obtained from orange and lemon peels at 100 °C for 8 min significantly affected the number of strains susceptible by significantly reducing the growth rate and lag phase.

The results obtained in this work support previous findings linking the growth inhibition of LEO against the two studied bacteria to its polyphenolic composition (mainly limonene, p-cymene and β-pinenes). In this sense, terpenes, terpenoids and other aromatic and aliphatic constituents with low molecular weights present in LEO could interact with the lipids of the cell membrane. This interaction could improve the membrane permeability, disturbing the cell’s structure and causing homeostasis, which is known as the state of steady internal physical and chemical conditions maintained by living systems, and it results in the leakage of ions and cytoplasmic content [48].

## 4. Conclusions

A new cascading approach to valorise lemon peels waste was successfully applied to, firstly, extract the essential oil using MAHD, obtaining a yield of 2.03% ± 0.21%. The water-to-waste ratio, energy and time used were optimised to minimise the energy consumption and promote environmentally sustainable extraction. Once the essential oil was obtained, the extraction of the pigment was optimised by MAE using a BBD design, obtaining an extract with a high content of flavonoids and a yellow colour at optimal conditions of 80% (*v*/*v*) ethanol, 80 °C and 50 min. Extraction yields of 5.9 ± 0.3 wt.% and final colour intensities of 0.669 ± 0.004 were obtained for LP. The composition of the essential oil was analysed by GC-FID, and the presences of limonene (65.082 wt.%), β-pinene (14.517 wt.%) and γ-terpinene (9.743 wt.%) were identified as characteristic compounds. The pigment was subjected to purification through adsorption resins and further analysis by UPLC-DAD-MS. The final purities of eriocitrin, diosmin and hesperidin were 4.728 wt.%, 7.368 wt.% and 2.658 wt.%, respectively, which corresponded to enrichment factors of 4.3, 7.4 and 5.0, respectively, by using Amberlite XAD16N resin. Finally, the essential oil showed strong inhibition against *E. coli* and *S. aureus* bacteria. In conclusion, the results obtained in this work showed the potential of microwave-assisted processes to effectively valorise lemon waste as a natural source of yellow pigments and antimicrobial flavours, contributing to the circular economy. A further scale-up of the proposed cascade method would contribute to reducing the high amounts of generated lemon peels wastes while obtaining functional ingredients to be used in different important industrial sectors such as food, cosmetic, medical and biocomposites.

## Figures and Tables

**Table 1:** Extraction conditions and obtained yield for lemon essential oil (LEO) from lemon waste by microwave-assisted hydrodistillation (MAHD). Mean ± Standard Deviation (SD), *n* = 3. Different superscripts in yield values indicate statistically significant different values (*p* < 0.05).

Conditions	Water-to-Waste Ratio (mL/g)			
	0.3 (No Agitation)		1 (Magnetic Stirring)	
	1st Step (Heating)	2nd Step (Extraction)	1st Step (Heating)	2nd Step (Extraction)
Irradiation power (W/g)	1.2	0.7	2.4	1.2
Extraction time (min)	5	10	5	10
Yield (wt.%)	2.03 ± 0.21 ^a^		1.91 ± 0.15 ^a^	

**Table 2:** Independent variables and selected levels used in the Box-Behnken design (BBD) for lemon pigment (LP) extraction from lemon peels.

Factors	−1	0	+1
Ethanol concentration (%, *v*/*v*)	20	50	80
Temperature (°C)	40	60	80
Extraction time (min)	5	32.5	60

**Table 3:** Physical properties of the adsorption resins used for LP (lemon pigment) purification.

Resin	Matrix	Polarity	Surface Area (m^2^/g)	Mean Pore Diameter (Å)
Amberlite XAD4	styrene-divinylbenzene	Nonpolar	750	100
Amberlite XAD7HP	acrylic	Polar	500	400
Amberlite XAD16N	styrene-divinylbenzene	Nonpolar	800	200

**Table 4:** Chemical composition of LEO fractions (wt.%) from lemon peels by MAHD. Mean ± SD, *n* = 3.

Compound	Retention Time (min)	Concentration (wt.%)
α-Thujene	8.75	0.461 ± 0.049
**α-Pinene**	**9.02**	**1.992** ± 0.081
**Sabinene**	**10.62**	**2.395** ± 0.104
**β-Pinene**	**10.81**	**14.517** ± 0.338
**Myrcene**	**11.35**	**1.427** ± 0.078
α-Terpinene	12.55	0.224 ± 0.060
p-Cymene	12.88	0.530 ± 0.010
**Limonene**	**13.16**	**65.082** ± 0.488
**γ-Terpinene**	**14.40**	**9.743** ± 0.087
Terpinolene	15.66	0.433 ± 0.015
Linalool	16.38	0.161 ± 0.033
Terpinen-4-ol	20.13	0.168 ± 0.064
α-Terpineol	20.82	0.272 ± 0.010
Nerol	22.25	0.169 ± 0.008
Neral	22.83	0.383 ± 0.018
Geranial	24.19	0.492 ± 0.060
Neryl acetate	28.26	0.400 ± 0.032
Geranyl acetate	29.12	0.273 ± 0.081
trans-Caryophyllene	30.86	0.228 ± 0.005
α-trans-Bergamotene	31.47	0.289 ± 0.011
β-Bisabolene	34.56	0.361 ± 0.009

In bold compounds with concentrations higher than 1 wt.%.

**Table 5:** Antimicrobial performance of LP and LEO against *Staphylococcus aureus* and *Escherichia coli*. Results are shown from triplicate samples after an initial inoculum size of 5.72 ± 0.26 and 5.80 ± 0.01 log colony-forming units (CFU)/mL, respectively. Mean ± SD, *n* = 3. Different superscripts in the same column values for each lemon additive indicate statistically significant different values compared to the control (*p* < 0.05).

	Extract Concentration (ppm)	*S. aureus* (log CFU/mL)	*E. coli* (log CFU/mL)
Control	-	7.69 ± 0.09 ^a^	7.50 ± 0.02 ^a^
LP	1000	7.40 ± 0.13 ^a^	7.62 ± 0.11 ^a^
	10,000	7.79 ± 0.09 ^a^	7.43 ± 0.27 ^a^
	100,000	7.96 ± 0.32 ^a^	7.78 ± 0.13 ^a^
LEO	15	7.05 ± 0.11 ^b^	>7.5 ^a^
	50	6.76 ± 0.74 ^b^	7.49 ± 0.09 ^a^
	150	5.54 ± 0.54 ^c^	6.09 ± 0.44 ^b^
	500	4.18 ± 0.91 ^c^	4.07 ± 1.93 ^c^
	1500	2.92 ± 1.05 ^d^	2.95 ± 1.09 ^cd^
	5000	<1 ^e^	1.45 ± 0.64 ^d^

## Supplementary Materials

The following are available online at https://www.mdpi.com/2304-8158/9/10/1493/s1: Table S1: BBD design matrix and response values obtained for LP extraction from lemon waste; Table S2: ANOVA results for response surface quadratic models of LP extraction.
