# Supplementary material for: Optimisation of Sequential Microwave-Assisted Extraction of Essential Oil and Pigment from Lemon Peels Waste
Source: Foods. 2020 Oct 19;9(10):1493. doi: 10.3390/foods9101493 (PMC7603390; doi:10.3390/foods9101493)
Supplement: Supplementary file 1 [file foods-09-01493-s001.pdf]

Table S1. BBD design matrix and response values obtained for LP extraction from lemon waste.

Run	Ethanol concentration (%, v/v)	Temperature (°C)	Extraction time (min)	Yield (%)	Colour intensity
1	50	60	32.5	4.2	0.409
2	20	80	32.5	3.2	0.299
3	50	80	5.0	5.4	0.441
4	80	60	60.0	6.2	0.412
5	50	40	5.0	4.5	0.411
6	50	60	32.5	3.8	0.405
7	50	40	60.0	5.3	0.376
8	50	80	60.0	5.6	0.555
9	20	60	60.0	4.4	0.369
10	20	40	32.5	4.7	0.423
11	50	60	32.5	3.9	0.413
12	80	40	32.5	4.9	0.497
13	80	80	32.5	5.1	0.664
14	20	60	5.0	3.6	0.336
15	80	60	5.0	4.3	0.476

Table S2. ANOVA results for response surface quadratic models of LP extraction.

		Colour intensity				Extraction yield			
Source	*Df*	*Sum of squares*	*Mean square*	*F-value*	*P-value*	*Sum of squares*	*Mean square*	*F-value*	*P-value*
A: Ethanol concentration	1	0.0484	0.0484	3022.53	0.0003***	2.6450	2.6450	61.04	0.0160***
B: Temperature	1	0.0079	0.0079	496.13	0.0020***	0.0012	0.0012	0.03	0.8808
C: Extraction time	1	0.0003	0.0003	18.00	0.0513	1.7112	1.7112	39.49	0.0244***
AA	1	0.0002	0.0002	11.72	0.0758	0.0041	0.0041	0.09	0.7874
AB	1	0.0212	0.0212	1323.14	0.0008***	0.7225	0.7225	16.67	0.0551
AC	1	0.0024	0.0024	147.02	0.0067***	0.3025	0.3025	6.98	0.1184
BB	1	0.0110	0.0110	688.59	0.0014***	1.0833	1.0833	25.00	0.0377***
BC	1	0.0056	0.0056	346.89	0.0029***	0.0900	0.0900	2.08	0.2863
CC	1	0.0012	0.0012	73.73	0.0133***	1.7664	1.7664	40.76	0.0237***
Lack-of-fit	3	0.0131	2.8833	3.08	0.1530	1.1625	0.3875	8.94	0.1022
Pure error	2	0.00003	0.9370			0.0867	0.0433		
Cor total	14	0.1117				9.4293			
R^2		0.8820				0.8675			

*** Significant at $p \leq 0.001$; ** significant at $p \leq 0.01$; * significant at $p \leq 0.05$.
